# Evaluation of a newly developed media-supported 4-step approach for basic life support training

**DOI:** 10.1186/1757-7241-20-37

**Published:** 2012-05-30

**Authors:** Saša Sopka, Henning Biermann, Rolf Rossaint, Sebastian Knott, Max Skorning, Jörg C Brokmann, Nicole Heussen, Stefan K Beckers

**Affiliations:** 1 Department of Anaesthesiology, Aachen, Germany; 2 Department of Intensive Care Medicine and Intermediate Care, Aachen, Germany; 3 Department of Medical Statistics, University Hospital Aachen, RWTH University, Pauwelsstr. 30, Aachen, Germany; 4 Department of Anaesthesiology, Section Emergency Medical Care, University Hospital Aachen, RWTH Aachen University, Pauwelsstr. 30, D–52074, Aachen, Germany

**Keywords:** Basic Life Support (BLS), Cardiopulmonary resuscitation (CPR), External chest compressions (ECC), Training, Media, 4-step approach

## Abstract

**Objective:**

The quality of external chest compressions (ECC) is of primary importance within basic life support (BLS). Recent guidelines delineate the so-called 4“-step approach” for teaching practical skills within resuscitation training guided by a certified instructor. The objective of this study was to evaluate whether a “media-supported 4-step approach” for BLS training leads to equal practical performance compared to the standard 4-step approach.

**Materials and methods:**

After baseline testing, 220 laypersons were either trained using the widely accepted method for resuscitation training (4-step approach) or using a newly created “media-supported 4-step approach”, both of equal duration. In this approach, steps 1 and 2 were ensured via a standardised self-produced podcast, which included all of the information regarding the BLS algorithm and resuscitation skills. Participants were tested on manikins in the same mock cardiac arrest single-rescuer scenario prior to intervention, after one week and after six months with respect to ECC-performance, and participants were surveyed about the approach.

**Results:**

Participants (age 23 ± 11, 69% female) reached comparable practical ECC performances in both groups, with no statistical difference. Even after six months, there was no difference detected in the quality of the initial assessment algorithm or delay concerning initiation of CPR. Overall, at least 99% of the intervention group (n = 99; mean 1.5 ± 0.8; 6-point Likert scale: 1 = completely agree, 6 = completely disagree) agreed that the video provided an adequate introduction to BLS skills.

**Conclusions:**

The “media-supported 4-step approach” leads to comparable practical ECC-performance compared to standard teaching, even with respect to retention of skills. Therefore, this approach could be useful in special educational settings where, for example, instructors’ resources are sparse or large-group sessions have to be prepared.

## Introduction

Within cardiopulmonary resuscitation (CPR), external chest compressions (ECC) are the key element providing forward blood flow, thereby maintaining heart and brain viability. Thus, ECC are of primary importance for improving outcomes after in- or out-of-hospital cardiac arrest.

Existing data about the ideal training methods for basic life support (BLS) are still insufficient; the duration of BLS training that is adequate remains unknown. Pedagogic training methods are thought to be the best, but specific auxiliary training methods that would improve laypersons and professionals performance have not been clearly identified. The standard method used in resuscitation training is the so-called “4-step approach” (syn. “4-stage approach”). This methodology was established based on the work of Peyton and was adapted by Bullock [1] for courses taught by the Resuscitation Council of the United Kingdom. This methodology was implemented as a basic principle of various course formats by the European Resuscitation Council (ERC); therefore, it is taught in all generic instructor courses [1-4]. However, there is no evidence that this approach leads to superior learning outcomes compared to other teaching methods. For this reason, recently published work is analysing the importance of the different steps [5] and their general performance as a whole with respect to skills acquisition [6].

Given that these educational concepts must be taught under special circumstances, including a lack of instructor resources and the necessity to teach large group sessions, feasible methods have to be available. An approach that uses up-to-date media to support both the instructor and the learner and that offers standardised content with integrated learning goals could be one such method.

The primary objective of this study was to evaluate and compare a new training method to the well-known four-step approach. This study investigates whether a “media-supported 4-step approach” (MSA) for BLS training initially leads to practical performance as good as the typically used 4-step-approach. Furthermore, the acquired skills were evaluated after one week and after six months concerning the same outcome parameter and the training participants’ acceptance of the new methods.

## Materials and methods

### Trial design

**Figure 1:**
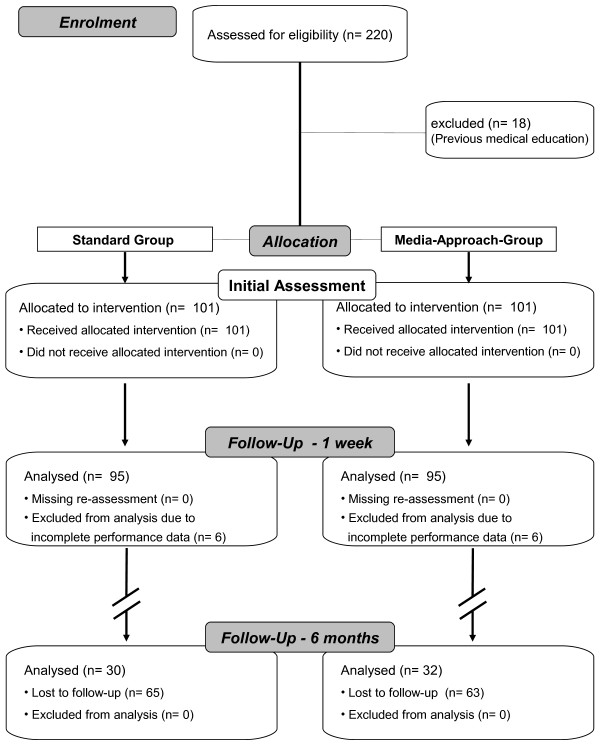
Overview study-design.

This controlled parallel-group trial was conducted at the Department of Anaesthesiology, University Hospital of Aachen, Germany, from 2008–2009 (Figures 1).

A randomisation procedure was not applicable because of the predefined group assignments within the Medical School organisation. The allocation of students to these groups was conducted by a representative of the Bureau of Student Affairs blinded to the study. The baseline testing was provided to ensure that there were no significant differences in the baseline characteristics between groups.

### Participants

Overall, 220 first-year medical students were asked to take part in the study during their initial weeks at the Medical School of the Rheinisch-Westfälische Technische Hochschule (RWTH) Aachen University, Aachen, Germany. The participants were all laypersons without previous medical training, professional CPR knowledge or CPR skills. Students who had completed any medical emergency training, such as that needed to become an emergency medical technician or a paramedic, before the beginning of this study, were excluded.

All students were informed that their performance would be evaluated and used for scientific purposes. The gender, age, weight and height of each student were collected, and informed consent was taken from each person prior to enrolment (enrolment details Figures 1). However, they were blinded and were not informed about the different learning approaches or their group allocation, and their individual evaluators were also blinded to the respective training approaches.

### Interventions

**Figure 2:**
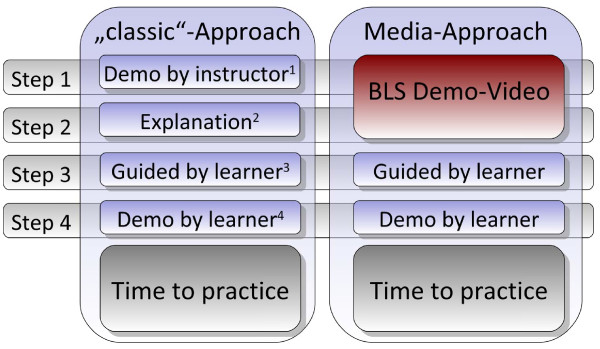
**Methodology of the Media-Supported 4-Step-Approach compared to the standard training approach**[1]**.** 1: Demonstration Phase; 2: Deconstruction Phase; 3: Comprehension Phase; 4: Performance Phase.

The performance of all participants was evaluated on a manikin in a mock cardiac arrest scenario before they were allocated to one of the study groups (comparison of approaches, Figures 2):

· Standard-group (S): Received BLS training with a standardised and common teaching methodology: the "4-step approach" (1;4).

· Group MSA: Received the newly created “media-supported 4-step approach” of the same course duration. In this approach, steps 1 (demonstrated by the instructor in real time) and 2 (explained in detail) were ensured via a standardised self-produced podcast (video/audio), which included all key information about the BLS algorithm and procedural resuscitation skills.

All tutorials in BLS, regardless of group assignment, were supervised by certified Advanced Life Support instructors from the ERC in the same manner. Every participant had the same time to train and the same support during training.

### Measurements, data acquisition

Participants were tested prior to the course, after one week and after six months following the initial BLS training. Each participant was individually evaluated following a standardised testing protocol and was not able to see other participants’ performances. The procedure was always performed as single-rescuer CPR (ECC in combination with mouth-to-mouth ventilation) and was terminated after up to 180 s duration.

The practical test setup consisted of a manikin (Skillreporter Resusci® Anne, Laerdal-Norway) connected to the Laerdal PC SkillReporting Software (Version 1.3.0, Laerdal-Norway) for data acquisition of applied compressions. The manikin was placed in a supine position on the floor and was dressed in a zippered jacket.

### Outcomes

#### Performance data

The observed primary outcomes were evaluated based on adaptations of the current guidelines [7]. These outcomes included the following: an average compression depth between 38 and 51 mm and a targeted rate of ECC between 90 and 110/min. This rate refers to the rate of compressions, not the absolute amount of compressions obtained per minute because of interruptions for ventilation. In addition, the total number of compressions without error, meaning those compressions that were of correct depth and had adequate release, was observed, and the percentage of incomplete releases between compressions – either too shallow or too deep – was also recorded.

A standardised checklist was used to compare the overall sequence of the BLS algorithm, whereas data were calculated for more than 60% correctness of the algorithm. Furthermore, a delay in the initiation of CPR was documented for each test.

#### Questionnaires

Self-confidence in CPR-performance was self-assessed before and after the intervention by each participant on a 10-point Likert scale (from 1 = completely unconfident to 10 = completely confident) for three items (BLS algorithm, mouth-to-mouth ventilation and ECC performance).

The participants allocated to the MSA group were also asked to evaluate whether the skills presented in the video were explicit, whether the video provided sufficient explanations and whether they would have preferred a lecture concerning the topic (6-point Likert scale: 1 = completely agree to 6 = completely disagree).

### Statistical analysis

Our primary outcome was the proportion of subjects who achieved targets for compression depth and compression rate. Based on a previous study [8], we estimated that with approximately 90 subjects per group, the study would have 80% power to show a greater rate of compression with correct depth in the media-approach group compared to the standard group (i.e., improvement from 51% in the standard group to 73% in the media-approach group) with a two-sided significance level of 5%. On the basis of this sample size, we calculated that we would have greater than 80% power to detect a difference in compressions with correct rates between the two groups (30% in the standard group to 83% in the media-approach group).

Depending on the scale of the endpoint, two-way repeated measures analysis of variance (repeated measures ANOVA) or two-way repeated logistic regression was calculated to investigate the effect of group (group factor, two levels: standard, media approach), time (group factor, 3 levels: baseline, after one week, after six months) and the resulting two-factor interaction of group and time. Suitable contrasts were formulated and tested to compare effects at a point of time within groups or between time points for one group. Data from the questionnaires were analysed using a paired *t*-test to compare the changes in self-assessed confidence scores before and after BLS training. An unpaired *t*-test was used to compare differences between groups.

Continuous variables were summarised by means and corresponding standard deviations (±). Categorical data were presented as percentages. All tests were two-sided and were assessed at the 5% significance level. Because of the exploratory nature of the parallel study hypotheses, we made no adjustment to the significance level to account for multiple tests. The analyses were performed using SAS® statistical software, V 9.2 (SAS Institute, Cary, NC, USA).

## Results

### Study population

Overall, 18 participants were excluded prior to the study because of a history of medical education (emergency medical technicians or paramedics). The demographic data of the participants were comparable between study groups (Table 1).

**Table 1:** Overview study population and demographics

**Qualification**	**Standard group**		**Media-sup. group**			
	**n**	**%**	**n**	**%**	**n**	**%**
Male	29	**28.7**	33	**32.7**	62	**30.7**
Female	72	**71.3**	68	**67.3**	140	**69.3**
Total	101		101		202	
**Demographics**					**p-value**	
Age	21.1 ± 3.8		21.6 ± 3.4		0.271	
Size (cm)	174.8 ± 10.9		171.3 ± 15.5		0.293	
Body weight (kg)	69.3 ± 15.6		67.2 ± 10.0		0.497	

Overview study population and demographics. p-value indicates differences between groups.

### Observed outcome data

#### Practical performance

**Figure 3:**
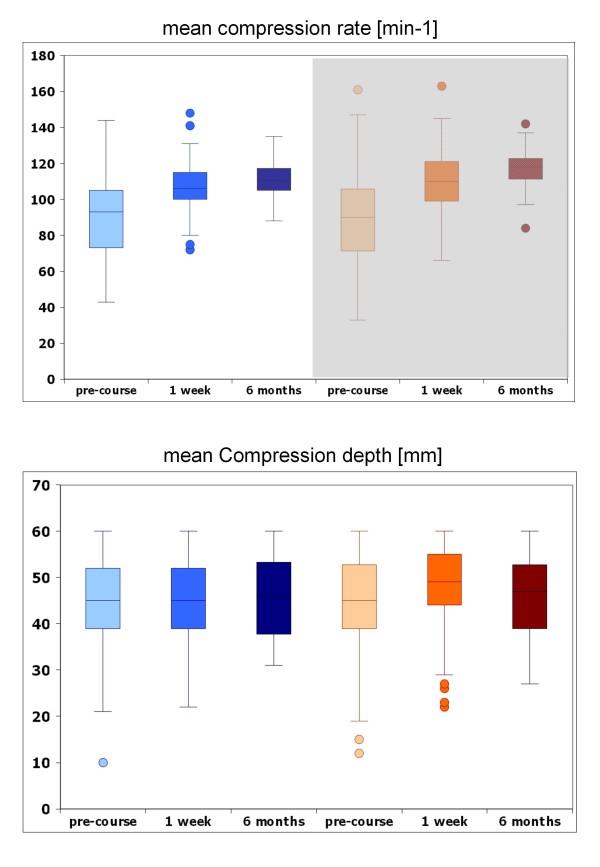
**Horizontal bars show the median, upper and lower end of the box representing 1st and 3rd quartile.** Narrow lines above and below the box represent the spread of values. Circles for values with 1.5–3-fold interquartile distance.

Both groups improved between the pre-course evaluation and the one-week evaluation, and they demonstrated comparable practical ECC performance (Table 2, Figures 3) with no statistical difference. In the MSA group, significantly more excessively deep compressions (p = 0.0354) and significantly fewer shallow compressions (p = 0.0252) were registered after one week, with no difference after six months compared to the standard group. Overall, ECC performance was still comparable in both groups, even after six months.

**Table 2:** Overview results performance

	**Standard**		**MSA**		**p-value**
**Baseline**	**(n = 95)**		**(n = 95)**		
	**n**	**%**	**n**	**%**	
**Compression rate 90–110/min**	35	**36.84**	28	**29.47**	0.2815
**Compression depth 38–51 mm**	51	**53.68**	45	**47.37**	0.3843
**No delay to start CPR**	55	**57.89**	64	**68.82**	0.1187
**Initial Assessment > 60% correct**	22	**23.16**	29	**30.53**	0.2530
**Compression without faults**	**33.49 ± 32.75**		**25.41 ± 32.80**		0.0795
**Compression too deep**	**25.53 ± 36.98**		**30.40 ± 40.58**		0.4214
**Compression too shallow**	**26.13 ± 34.96**		**26.34 ± 38.16**		0.8410
**After 1 week**	**(n = 95)**		**(n = 95)**		
**Compression rate 90–110/min**	45	**47.37**	38	**40.00**	0.3064
**Compression depth 38–51 mm**	46	**48.42**	48	**50.53**	0.7717
**No delay to start CPR**	80	**84.21**^$^	84	**88.42**^$^	0.4001
**Initial Assessment > 60% correct**	81	85.26*	87	91.58*	0.1787
**Compression without faults**	**39.75 ± 34.44**		**36.50 ± 35.02**^**†**^		0.5170
**Compression too deep**	**28.52 ± 37.17**		**40.56 ± 41.49**^**¥**^		**0.0354**
**Compression too shallow**	**24.45 ± 34.05**		**14.07 ± 29.55**^‡^		**0.0252**
**After 6 months**	**(n = 30)**		**(n = 32)**		
**Compression rate 90–110/min**	13	**43.33**	7	**21.88**	0.3425
**Compression depth 38–51 mm**	14	**46.67**	18	**56.25**	0.7695
**No delay to start CPR**	27	**90**^**£1**^	26	**83.87**^**£2**^	0.5195
**Initial Assessment > 60% correct**	25	**83.33**^**#**^	26	**81.25**^**#**^	0.9330
**Compression without faults**	**40.20 ± 32.08**		**38.69 ± 34.44**		0.7401
**Compression too deep**	**28.46 ± 37.82**		**32.75 ± 38.03**		0.5860
**Compression too shallow**	**24.57 ± 31.75**		**20.41 ± 31.77**		0.9477

Results of performance data for the different study groups in the three evaluations. Data in x = n and percentage of respective group size or in absolute numbers ± standard deviation.

Time to start CPR in seconds. Compression without faults: adequate compression depth and correct chest well decompression. Significant differences between groups are indicated **bold**.

Significant differences between time-points:

* baseline = > 1 week: p < 0.0001; ^$^ baseline = > 1 week: p = 0.0003; † baseline = > 1 week: p = 0.0089;

‡ baseline = > 1 week: p = 0.006; ¥ baseline = > 1 week: p = 0.0072.

^#^ baseline = > 6 months: p < 0.0001; ^£1^ baseline = > 6 months: p < 0.0014; ^£2^ baseline = > 6 months: p < 0.0496.

One week after intervention, both groups improved concerning the quality of the initial assessment algorithm (p < 0.0001), which was detectable even after a period of six months (p < 0.0001). Post-course, more than 85% of the participants in both groups demonstrated no delay in the initiation of CPR (improvement of standard group: p < 0.0001; MSA: p = 0.0003; Table 2). When comparing demographic to performance data, the groups were not statistically different.

#### Questionnaires

Overall, 202 questionnaires were collected in the evaluation of course assessment. With regard to the questions related to the quality and content of the BLS video, 46.5% (n = 46) of the participants agreed completely (36.4%; n = 36 agreed) that the demonstration of the BLS skills was “explicit” (mean ± SD: 1.7 ± 0.8). Overall, 59.6% (n = 59) agreed completely (30.3%; n = 30 agreed) that the video provided sufficient explanations of the topic (1.5 ± 0.8). In contrast, only 1% (n = 1; 5.2 ± 0.9) would have preferred a lecture on the topic instead of a video.

Regarding participant self-confidence data from over 120 questionnaires, no significant differences between participants from the two groups were identified with respect to knowledge of the BLS algorithm or ECC performance before or after training: BLS algorithm before (S: 4.6 ± 2.4 vs. MSA: 4.6 ± 2.5; p = 0.997) and after training (S: 7.9 ± 2.1 vs. MSA: 8.1 ± 1.8; p = 0.675); external chest compressions before (S: 4.7 ± 2.7 vs. MSA: 4.9 ± 2.3; p = 0.721) and after training (S: 7.8 ± 2.2 vs. MSA: 8.1 ± 1.7; p = 0.433).

Nevertheless, there were significant improvements in the self-confidence levels in both groups before and after training (Group S: BLS algorithm from 4.2 ± 2.5 to 7.9 ± 2.2 (p < 0.0001); Group S: ECC performance from 4.7 ± 2.8 to 7.8 ± 2.3 (p < 0.0001); Group MSA: BLS-algorithm 4.7 ± 2.6 to 8.1 ± 1.8 (p < 0.0001); Group MSA: ECC performance 5.0 ± 2.6 to 8.1 ± 1.7 (p < 0.0001)).

## Discussion

To evaluate alternative teaching methods for BLS, we performed a study comparing the widely practised and accepted 4-step approach with the newly developed media-supported approach (MSA) in a group of laypersons. This controlled trial tested if the new approach could achieve comparable performance concerning the acquisition of BLS skills.

The main result of this study is that the MSA for resuscitation training compared to the established methodology leads to comparable retention of skills both one week after the training and six months later. Both educational approaches result in comparable practical performance. Compared to baseline testing, the one-week evaluation showed a significant improvement for various parameters, including correctness of the initial assessment algorithm and no delay in CPR initiation; additionally, no significant differences between groups were identified for the mean compression depth and mean compression rate. Compared to the standard group, after one week, the participants in the MSA group had significantly more compressions that were “too deep” and fewer that were “too shallow”. It is promising that the MSA group tended to have deeper as opposed to shallower compressions. Hopefully, this fact will result in superior performance according to the newly recommended range for compression depth (50 to 60 mm instead 38 to 51 mm) in the 2010 ERC Guidelines [9]. After six months, the MSA group did not exhibit significantly different results concerning practical performance data. In fact, correctness of the initial assessment algorithm (>60% correct) tended to be recalled better in this group (S: 83% vs. MSA: 81%; p = 0.933); the compression depth was also superior (S: 47% vs. MSA: 56%; p = 0.7965), but the difference was not statistically significant.

Furthermore, the participants reported significant improvements in self-confidence concerning the BLS algorithm and ECC performance in both groups. There were no significant differences in the self-confidence levels of participants regarding the observed skills between the groups at any time.

Our findings are consistent with other studies that have investigated using video-based teaching in CPR. Lynch et al. reported that a 30-min self-instruction program enables laypersons to become as good at performing CPR as laypersons trained with an instructor [10].

The high efficiency of this newly established MSA training method is based not only on equivalent practical skills of ECC but also on results regarding the individual self-assessments of participants between the groups, which were also equivalent. Analysis of BLS training needs to be undertaken from different points of view; thus, a 360-degree investigation should be the final goal.

Studies regarding the willingness of laypersons to perform bystander CPR with prior BLS training [11] confirm this finding. Similar studies have reported that laypersons with prior BLS training were more confident with CPR initiation [12] and were more willing to perform bystander CPR [13,14]. According to this finding, the MSA training method may lead to the same bystander rate as the traditional 4-step approach*.*

The typical 4-step approach method is widely accepted and used by various organisations that teach, similar to the ERC (1;2;4) or the American Heart Association [3]. This approach is dependent on qualified instructors who need to be taught and certified by extensive procedures. Not every organisation, company or medical institution has sufficient resources to provide enough instructors for teaching large groups of laypersons or medical staff. In addition, insecurity and variation in the quality and standardisation of the instructors will always exist. Importantly, almost 20 years ago, the instructor was identified as one possible reason for the poor retention of skills [15]. The modified “media-supported 4-step approach” could represent one possibility for enhancing the range of training methods. Usage of media, including video, offers different capabilities for involving the learner in a self-directed learning process as part of a blended learning concept. Bobrow et al. showed that usage of an ultra-short hands-only CPR video improves CPR practical skills. This investigation revealed evidence that simply the usage of short educational videos has an impressive effect on the acquisition of ECC skills [16]. If new technologies are used, such as videos or portable devices, learners can refresh themselves as often as needed. Our results regarding the retention of skills and usage of video-based teaching/training show that this tool could be useful and effective. In addition, Einspruch et al. showed that the retention of ECC skills is poor two months after training [17]. It is possible that self-instructed video-based training could be very useful for refreshing important CPR skills.

Based on these results, it seems worthwhile to establish a concept that integrates videos for more self-directed BLS learning. Different study groups demonstrated that teaching methods, such as self-directed learning using video-based BLS self-instruction, is an appropriate approach for special groups of learners [18-20]. A study by Nishiyama et al. showed that watching a CPR instruction video as a self-directed learning tool encouraged people to perform CPR and to use an automatic external defibrillator. Moreover, the study showed that practical training in BLS is indispensable [8].

Further investigation should include the implementation of interactive learner software installed on portable devices. Methods such as MSA could support the shift from teacher-centred learning to learner-centred course concepts. However, there is no evidence that the typical 4-step approach leads to superior learning outcomes. Recently published work by Greif et al. [7] showed no advantage for skills acquisition compared to a traditional 2-step approach, as embodied by the “see one, do one” approach, for percutaneous needle-puncture cricothyroidotomy. Another study group also reported similar results in comparing the 4-step approach with a 2-step method for laryngeal mask insertion [9]. Indeed, it might be questionable if these results are transferable to a comparably more complex “skill” (i.e., ECC) within a complete algorithm such as BLS. In fact, Greif et al. postulated that “…further investigations in more complex skills under different settings might show the superiority of the 4-stage approach …”.[5]

Our results show that the investigated MSA method of teaching BLS, specifically as it pertains to ECC skills, is equivalent to the typical instructor-based 4-step approach. The quality of ECC performance was at the same level both after the training and six months later. Obviously, the MSA offers an additional alternative for teaching ECC among BLS skills, but more investigation into these findings is needed.

Furthermore, our study could demonstrate that this type of methodological approach is feasible and accepted by participants. Looking at the questionnaire data, the majority of participants belonging to the MSA group completely agreed that the BLS skills were demonstrated “explicitly” and that they were provided with sufficient explanations of the topic at hand. Only a minority would prefer to have a lecture on the topic.

However, more data need to be collected to determine how effective and efficient these methods are for improving the survival rate of patients with cardiac arrest.

### Limitations

Regarding possible limitations, the observed study group was not equally distributed with respect to gender (69% female), but the distribution was comparable between groups. Additionally, the participants were not chosen at random but rather represent inexperienced laypersons with regard to emergency medical issues because German medical students start medical school immediately after graduation from secondary school without any prior preparation. However, based on the reported baseline results, it is suitable to state that our participants did not differ; therefore, groups are comparable. Like previous studies, we argue that the participants are laypersons concerning CPR and are not representative of or similar to health-care professionals [21,22].

Furthermore, in this kind of simulation study, each manikin used is unable to mimic a human, especially when representing an unconscious, breathless and pulseless victim. Nevertheless, the use of manikins as a tool for testing is appropriate, and multiple studies have previously described relevant methodologies [23-28].

A methodical limitation might be the dropout rate by the six-month evaluation; the main reason for this is that many participants had other mandatory appointments during these evaluation periods. We are aware that, in general, voluntary participants might initiate a positive selection bias, such that these volunteers would often outperform others [29]. However, as described in the literature [30,31], all of our study groups were exposed to this bias; therefore, they are still comparable.

## Conclusion

Evaluation data clearly demonstrate the feasibility and acceptance of the “media-supported 4-step approach”. In addition, the proposed concept leads to comparable but not superior retention of skills and comparable levels of self-confidence after six months. Therefore, this approach might be used as an alternative to the traditional 4-step approach, especially to ensure standardisation of key topics or when instructor resources are sparse.

## Abbreviations

BLS: Basic life support; CPR: Cardiopulmonary resuscitation; ECC: External chest compressions; ERC: European resuscitation council; MSA: Media-supported approach.

## Competing interests

The authors declare that they have no competing interests.

## Authors´ contribution

SSO was responcible for conception and design, acquisition, analysis and interpretation of the data of this study. Furthermore he was drafting the manuscript. HB was involved in acquisition of the data analysis and interpretation of the data and in the critical revision of the manuscript for important intellectual content. RR was involved in the analysis and interpretation of the data and a critical recvision of the manuscript for important intellectual content. SK was responcible for the acquisition and interpretation of the data. MS was involved in the drafting of the manuscript and the critical revision for important intellectual content. JB was involved in the analysis and interpretation of the data and the critical revision of the manuscript. NH was responcible for the statistical analysis and the interpretation of the data. Furthermore she was involved in drafting the manuscript. SKB was responcible for the study concept and design. He was drafting the manuscript and involved in the interpretation of the data. Furthermore he revised the manuscript critically and was responcible for the study supervision. All authors read and approved the final manuscript.
